# Structure and Anti-Tumor Activities of Exopolysaccharides from *Alternaria mali* Roberts

**DOI:** 10.3390/molecules24071345

**Published:** 2019-04-05

**Authors:** Pingchuan Yuan, Fang Fang, Taili Shao, Ping Li, Wei Hu, Yuyan Zhou, Guodong Wang, Jun Han, Kaoshan Chen

**Affiliations:** 1 Anhui Provincial Engineering Research Center for Polysaccharide Drugs, Anhui Province Key Laboratory of Active Biological Macro-Molecules, Drug Research & Development Center, School of Pharmacy, Wannan Medical College, Wuhu 241000, China; 20180042@wnmc.edu.cn (P.Y.); fangfangyaoxue@sina.com (F.F.); shaotaili@wnmc.edu.cn (T.S.); liping919@wnmc.edu.cn (P.L.); mysteryzyy@163.com (Y.Z.); 2 Department of Medical Parasitology, Wannan Medical College, Wuhu 241000, China; huwei198703@163.com; 3 School of Life Science, National Glycoengineering Research Center, State Key Laboratory of Microbial Technology, Shandong University, Jinan 250100, China

**Keywords:** *Alternaria mali* Roberts, extracellular polysaccharide, structure, apoptosis

## Abstract

In this study, an extracellular polysaccharide from *Alternaria mali* Roberts (AMEP) was extracted, and its structure was characterized, in addition to its antitumor activity in vitro. Neutral polysaccharide AMEP-1 and anionic polysaccharide AMEP-2 were isolated from AMEP, and their monosaccharide compositions consisted of mannose (Man), glucose (Glc), and galactose (Gal) but at different ratios. The linking mode of both AMEP-1 and AMEP-2 is Man*p*-(1→4) and Glc*p*-(1→6), and the branched chains are connected to the main chain through O-6. AMEP-2 inhibited the proliferation of BGC-823 cells in a time- and concentration-dependent manner. AMEP-2 also induced the apoptosis of BGC-823 cells, and showed anti-tumor effects by inducing cell cycle arrest in the S phase, reactive oxygen species production, and mitochondrial membrane potential reduction in BGC-823 cells. Therefore, AMEP-2 shows potential for further development as a novel anti-tumor agent.

## 1. Introduction

Cancer remains a serious threat to human life and safety, and the conventional chemotherapy drugs used in cancer treatment have strong cytotoxicity, low selectivity, and induce damage to the host immune system, often resulting in serious infection to exert more damage to patients than the initial disease [1,2,3]. A study of the long-term screening of anticancer drugs revealed that many polysaccharide-based drugs have a remarkable effect with respect to anti-tumor activity and immunoregulation [4,5,6,7,8,9]. Polysaccharides can be used to fight tumors in many ways: they can exert cytotoxicity to kill cancer cells directly; suppress the invasion, adhesion, and metastatic ability of cancer cells; inhibit the growth of cancer cells; induce cell cycle arrest; activate mitochondria and death receptor pathways to induce the cell death processes of tumor cells; mediate signal transduction pathways; or influence the expression of oncogenes and tumor suppressor genes [10,11,12,13,14]. Moreover, the combination of polysaccharides and chemotherapeutic drugs has been shown to increase the sensitivity to chemotherapeutic drugs while simultaneously reducing their toxic side effects [15,16,17]. Therefore, further exploration of naturally found polysaccharides can advance the development of new cancer treatments and combination treatments to improve the outcome of chemotherapy and the quality of life of patients.

In long-term studies, several polysaccharides extracted from fungi, such as polysaccharides from *Grifolafrondosa* [18], *Ganodermalucidum* [19], *Gastrodiaelata* Blume [20], *Trichodermapseudokoningii* [6,21], and *Flammulinavelutipes* [22], have exhibited strong biological activity, especially anti-tumor and immunoregulatory activity [10,23,24,25,26]. We have been devoted to exploring polysaccharides with antitumor activity from various fungi for a long time [6,7,27,28,29]. *Alternaria mali* Roberts is a pathogenic fungus of the apple, which causes apple spot blight after infection. Herein, we found two kinds of polysaccharides, AMEP-1 and AMEP-2, after isolation and purification from the fermentation broth of *Alternaria mali* Roberts. This study aimed to characterize the structure of AMEP, evaluate its antitumor activity by human gastric cancer cell line BGC-823 in vitro, and conduct a preliminary exploration of the potential mechanism. The results of this study lay a foundation for the development of novel active polysaccharides, drugs, and functional foods for the prevention and treatment of cancer.

## 2. Results and Discussion

### 2.1. Purity and Molecular Weight of AMEP-1 and AMEP-2

**Figure 1:**
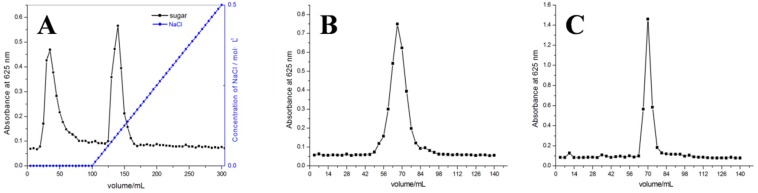
DEAE-FF and S-400 HR elution curves of the AMEPs: (**A**) DEAE-FF elution curves of the AMEP; (**B**) S-400 HR elution curves of the AMEP-1; and (**C**) S-400 HR elution curves of the AMEP-2.

Crude polysaccharides were separated on a DEAE-FF (DEAE-Sepharose Fast Flow) ion-exchange chromatography column. The components corresponding to the two peaks were collected according to the elution curve, and the two polysaccharides were named AMEP-1 and AMEP-2, respectively. S-400 HR gel chromatography columns were further used for purification of the polysaccharides using distilled water (Figure 1). Pure polysaccharides were obtained by lyophilization after 48 h of dialysis using pure water. Purified AMEP-1 and AMEP-2 appeared as white powders and showed a negative tri-iodide reaction, indicating the absence of a starch-type polysaccharide. Moreover, Coomassie brilliant blue G-250 staining of AMEP-1 and AMEP-2 protein showed no obvious color reaction.

**Figure 2:**
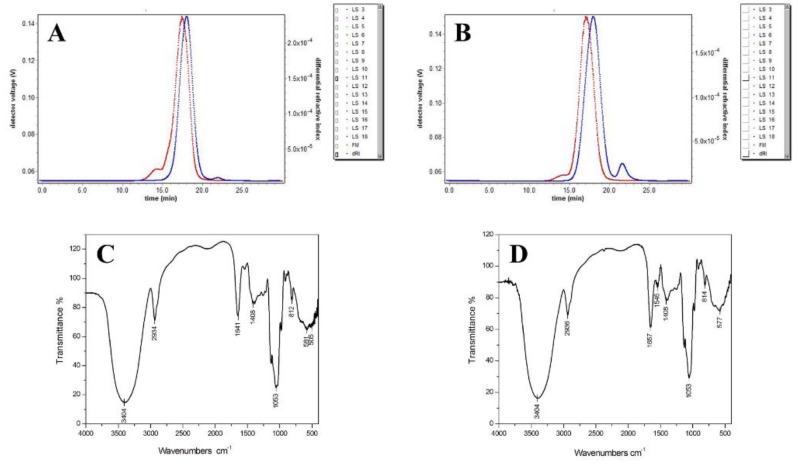
HPSEC-MALLS chromatographic profiles and IR spectra ofAMEP-1 and AMEP-2: (**A**) HPSEC-MALLS chromatographic profile of AMEP-1; (**B**) HPSEC-MALLS chromatographic profile of AMEP-2; (**C**) IR spectrum of AMEP-1; and (**D**) IR spectrum of AMEP-2.

The HPSEC chromatogram (Figure 2A,B) showed a symmetrical shape with more than two polysaccharides components, and the polysaccharides AMEP-1 and AMEP-2 were clearly divided into uniform groups. As shown in Table 1, MALLS analysis of AMEP-1 and AMEP-2 revealed an average molecular weight of 3.861 × 10^4^ Da and 4.689 × 10^4^ Da, respectively. The dispersion indices of AMEP-1 and AMEP-2 were 1.492 and 1.849, respectively, confirming the relatively uniform structure.

### 2.2. Monosaccharide Composition of AMEP-1 and AMEP-2

**Figure 3:**
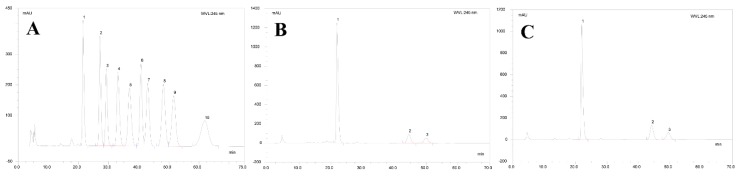
HPLC results of standard monosaccharides, AMEP-1 and AMEP-2: (**A**) HPLC results for standard monosaccharides (1-Man, 2-GlcN, 3-Rha, 4-GlcA, 5-GalA, 6-GalN, 7-Glc, 8-Gal, 9-Xyl, and 10-Fuc); (**B**) HPLC results of AMEP-1 monosaccharides composition (1-Man, 2-Glc, and 3-Gal); and (**C**) HPLC results of AMEP-2 monosaccharides composition (1-Man, 2-Glc, and 3-Gal).

HPLC was used to analyze the monosaccharides of the AMEP-1 and AMEP-2. HPLC resulted in good separation of each monosaccharide standard (Figure 3). After hydrolysis, AMEP-1 and AMEP-2 were analyzed under the same condition as the standard monosaccharide mixture. AMEP-1 and AMEP-2 contain Man, Glc, and Gal at a molar ratio of 12.9:1.6:1 and 9.1:2.0:1, respectively. Therefore, although the monosaccharides composition is the same for the two polysaccharides, their proportions are slightly different.

### 2.3. Infrared Spectra of AMEP-1 and AMEP-2

The infrared spectra showed that both AMEP-1 and AMEP-2 had characteristic absorptions peaks of polysaccharides (Figure 2C,D). Specifically, both AMEP-1 and AMEP-2 showed a wide and strong absorption peak at approximately 3400 cm^−1^, corresponding to an O-H stretching vibration peak, and another peak at 2930 cm^−1^ corresponding to the C-H stretching vibration peak, which are the characteristic absorption peaks of polysaccharides. In addition, the absorption peaks at 1641 cm^−1^ and 1657 cm^−1^ are the contractive vibration absorption of carbonyl C=O. The absorption peak at 1400 cm^−1^ is near the C-H variable angle vibration absorption peak, while that at 1200–1000 cm^−1^ is within the scope of the glycosidic link C-O-C stretching vibration and hydroxyl C-O-H variable angle vibration absorption peak. Moreover, the peaks at 812 cm^−1^ and 814 cm^−1^ are characteristic of mannose glucan, which is consistent with the results of the monosaccharide composition analysis. AMEP-2 was not absorbed at 1720 cm^−1^. The results were consistent with the analysis of monosaccharide composition. AMEP-2 did not contain uronic acid [30,31].

### 2.4. NMR Spectra of AMEP-1 and AMEP-2

In the ^1^H spectra, the δ3–4 ppm signal overlaps to parse ppm, with δ4.8–5.5 ppm representing the anomeric proton resonance region. The anomeric α glycosidic bond carbon chemical shifts are usually greater than δ5ppm, while the anomeric carbon signal type-β glycosidic bonds are usually smaller than δ5 ppm; the anomeric carbon signal is smaller than δ5.4 ppm in the pyranose type, but is greater than δ5.4 ppm for the furanose type [30,32,33]. 

**Figure 4:**
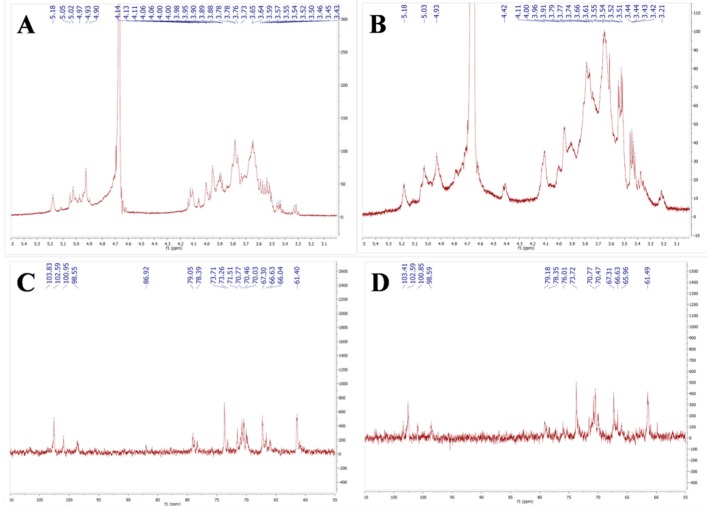
NMR spectra of AMEP-1 and AMEP-2: (**A**) ^1^H-NMR spectrum of AMEP-1; (**B**) ^1^H-NMR spectrum of AMEP-2; (**C**) ^13^C-NMR spectrum of AMEP-1; and (**D**) ^13^C-NMR spectrum of AMEP-2.

The ^1^H-NMR (500 MHz, D_2_O) signals of AMEP-1 and AMEP-2 are mainly concentrated in the range of δ3.0–5.5 ppm (Figure 4A,B). Among them, the δ4.3–5.5 ppm region is the main terminal group protons signal. The δ3.1–4.3 ppm region is the proton signal of the sugar ring.

^13^C-NMR (126 MHz, D_2_O) of AMEP-1 and AMEP-2 (Figure 4C,D): The signal of NMR carbon spectrum is mainly concentrated in the range of δ60–110 ppm. By observing the carbon spectrum, we found that the peak of the main anomeric carbon signal is within δ97–110 ppm. The main signal peaks are distributed in the region of δ60–85 ppm, in which δ86.92 ppm of AMEP-1 migrates to low field, indicating that C3 is replaced. Two signal peaks of δ79.05 ppm and δ78.39 ppm migrate to low field, indicating the existence of C4 substituted glycoside bonds. The four signal peaks of C6 are distributed in the high field region of δ60–70 ppm. The signal peaks of δ67.30, 66.63 and 66.04 ppm migrate to the low field, and C6 may be replaced. The signal peaks of AMEP-2 at δ79.18 ppm migrates to low field, indicating that there is a C4 substituted glycoside bond. The peak of C6 signal mainly distributes in the high field area of δ60–70 ppm. We observed a signal peak of δ61.49 ppm, which belongs to the signal peak of C6. The signal peaks of δ67.30, 66.63 and 65.96 ppm migrate in low field, and C6 may be replaced.

According to the analysis of monosaccharide composition, AMEP-1 and AMEP-2 are mainly composed of mannose, with a small amount of glucose and galactose. According to the abundance of carbon spectrum signal peak, we can deduce that AMEP-1 has the highest signal peak at the position of δ103.83 ppm and should be the signal of mannose anomeric carbon. The signal peak of mannose C2/4 was δ79.09 ppm, and the signal peak of C6 was δ61.4 ppm. In addition, the signal peak with high abundance of δ66.6 ppm was replaced. AMEP-2 has the highest signal peak of δ102.59 ppm, which should be mannose anomeric carbon signal, δ79.18 ppm, δ78.35 ppm which should be mannose C2/4 signal peak, and C6 signal peaks of δ65.96 ppm and δ61.49 ppm. Therefore, the possible links of AMEP-1 and AMEP-2 are Man*p*-(1→4) or Man*p*-(1→6), other links may be Man*p*-(1→2). Among them, the δ98.55 ppm of AMEP-1 may be the anomeric carbon signal of glucose, the δ86.92 ppm may belong to C3, and the δ66.04 ppm may belong to the C6 of glucose. The δ98.55 ppm of AMEP-2 may be the anomeric carbon signal of glucose, δ66.63 ppm may belong to C6 of glucose, so AMEP-1 and AMEP-2 may contain Glc*p*-(1→6). Because the galactose signal is relatively weak, it is difficult to distinguish and judge the corresponding signal peaks in 1D-NMR.

In summary, AMEP-1 and AMEP-2 are mainly mannan. The linking mode is Man*p*-(1→4) and Glc*p*-(1→6), the branched chains are connected to the main chain through O-6.

### 2.5. Effect of AMEP-2 on Proliferation of the Human Gastric Cancer Cell Line BGC-823

BGC-823 cells were incubated with different concentrations of AMEP-2 for 24, 48, and 72 h, and MTT assay was used to detect its effects on cell proliferation. When the concentration of AMEP-2 was increased from 200 to 2000 μg/mL, the cell inhibition rate increased proportionally, representing a statistically significant difference compared with the control group (Table 2). These results demonstrate that AMEP-2 could effectively inhibit the proliferation of BGC-823 cells in a time- and concentration-dependent manner.

### 2.6. Effect of AMEP-2 on the Cell Cycle in BGC-823 Cells

**Figure 5:**
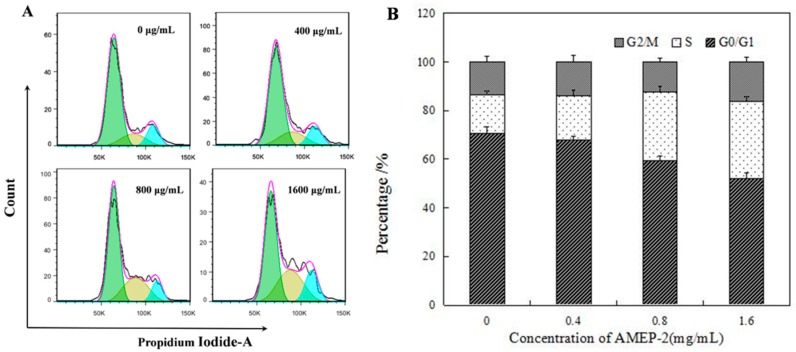
Effects of AMEP-2 on the cell cycle in BGC-823 cells: (**A**) Effects of AMEP-2 on the cell cycle in BGC-823 cells by Flow cytometry; (**B**) Percentage Chart of the Effects of AMEP-2 on the cell cycle in BGC-823 cells.

BGC-823 cells were treated with different concentrations of AMEP-2 (0, 400, 800, and 1600 μg/mL) for 48 h, and PI staining was performed followed by flow cytometry to detect the cell cycle. As shown in Figure 5, the percentage of cells in the G2/M phase was 13.69%, 13.76%, 12.44%, and 16.1% compared with that in the control group, and the proportion of cells in the S phase increased in a concentration-dependent manner at 15.74%, 18.16%, 27.97%, and 31.78%, respectively. Therefore, AMEP-2 could block cells in the S phase, resulting in abnormal division and proliferation. This suggests that induction of cell cycle arrest is a possible mechanism by which AMEP-2 inhibits the proliferation of BGC-823 cells.

### 2.7. Effect of AMEP-2 on the Apoptosis of BGC-823 Cells

**Figure 6:**
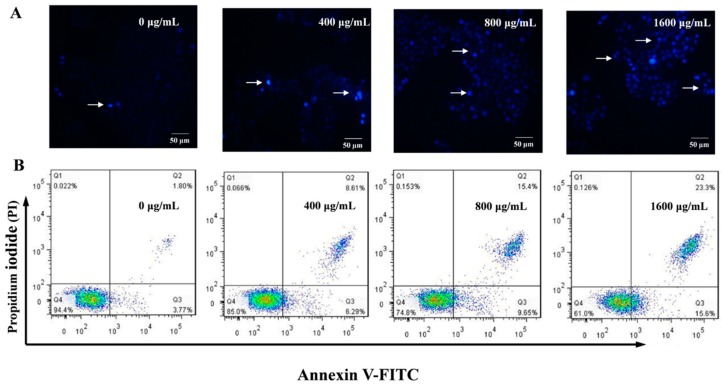
Effect of AMEP-2 on the apoptosis of BGC-823 cells: (**A**) Hoechst 33258 staining. BGC-823 cells were treated with 0, 400, 800 and 1600 μg/mL AMEP-2 for 48 h, Bar = 50 μm; and (**B**) Annexin V/PI dual staining of BGC-823 cells. BGC-823 cells were treated with 0, 400, 800 and 1600 μg/mL AMEP-2.

As shown in Figure 6A, the BGC-823 cells treated with different concentrations of AMEP-2 (400, 800, and 1600 μg/mL) for 48 h were stained with Hoechst 33258 and observed by fluorescence microscopy (arrows point to the apoptotic cells). The control group showed uniform nuclear staining with a clear and complete boundary, emitting pale blue fluorescence. However, after AMEP-2 treatment, the nuclei of the cells decreased, chromatin condensed, dense staining and bright blue fluorescence were produced, and some apoptotic bodies were also induced. Therefore, AMEP-2 could clearly induce the apoptosis of BGC-823 cells.

Apoptosis was also detected through an Annexin V-FITC/PI double-staining assay. As shown in Figure 6B, with an increase in AMEP-2 concentration, there was an increase in the rates of early apoptosis, late apoptosis, and necrosis in BGC-823 cells. At 400, 800, and 1600 μg/mL of AMEP-2, the ratio of early apoptotic cells was 6.29%, 9.65%, and 15.6%, respectively, and the ratio of late apoptosis and necrotic cells was 8.61%, 15.4%, and 23.3%, respectively.

### 2.8. Effect of AMEP-2 on ROS Generation in BGC-823 Cells

**Figure 7:**
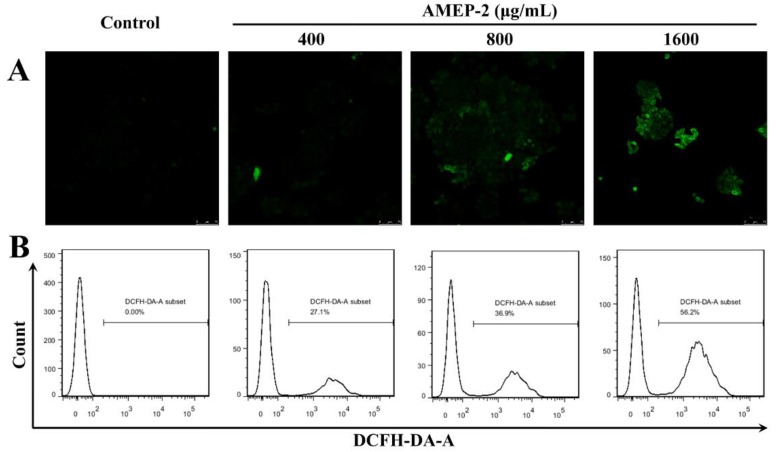
Effect of AMEP-2 on ROS production in BGC-823 cells: (**A**) Effect of AMEP-2 on ROS production in BGC-823 Cells by Immunofluorescence; (**B**) Effect of AMEP-2 on ROS production in BGC-823 Cells by Flow cytometry.

The intracellular reactive oxygen oxidation of DCF (dichlorofluorescein) can be detected by monitoring the change in fluorescence to reflect the intracellular ROS (reactive oxygen species) level. BGC-823 cells were treated with different concentrations of AMEP-2 (400, 800, and 1600 μg/mL) for 48 h and loaded with the DCFH-DA (2,7-dichlorodihydrofluorescein diacetate) fluorescent probe. The ROS content in BGC-823 cells was detected by flow cytometry and confocal laser-scanning microscopy. The results of flow cytometry showed that the number of ROS increased with an increase in AMEP-2 concentration to 27.1%, 36.9%, and 56.2%, respectively (Figure 7B). Confocal laser-scanning microscopy showed that the fluorescence of the cells in the control group was weak, whereas the overall fluorescence intensity of the cells increased continuously with an increase in AMEP-2 concentration, reflecting an increase in the intracellular ROS content compared to that in the control group (Figure 7A). The results show that AMEP-2 could increase the level of oxidative stress in BGC-823 cells and promote its apoptosis.

### 2.9. Effect of AMEP-2 on Mitochondrial Membrane Potential in BGC-823 Cells

**Figure 8:**
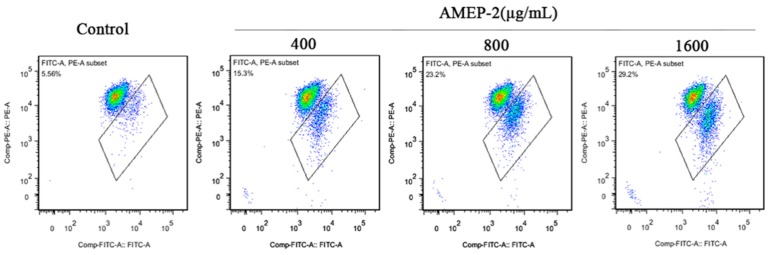
Effect of AMEP-2 on the mitochondrial membrane potential in BGC-823 cells.

A significant event of early apoptosis is the decrease in mitochondrial membrane potential. Mitochondria are the main producers of ROS, which in turn destroy the mitochondria to reduce mitochondrial membrane potential and ultimately induce apoptosis. The reduced ratio of red to green fluorescence indicates the disruption of mitochondrial membrane potential. In this study, we used JC-1 as an ideal fluorescent probe to detect changes in mitochondrial membrane potential. The BGC-823 cells were treated with different concentrations of AMEP-2 (400, 800, 1600 μg/mL) for 48 h and loaded with the JC-1 fluorescent probe, and the mitochondrial membrane potential changes in BGC-823 cells were detected by flow cytometry. Along with the increase of AMEP-2 concentration, the reduced ratio of red to green fluorescence was increased to 15.3%, 23.2% and 29.2%, respectively (Figure 8). Therefore, the mitochondrial membrane was destroyed as a result of AMEP-2 treatment.

## 3. Materials and Methods

### 3.1. Culture and Polysaccharide Production of A. Mali Roberts

*Alternaria mali* Roberts strain was isolated from an apple, which was collected from Shandong Province, China, and identified by Professor Qi Junshan (Shandong Academy of Agricultural Sciences, Jinan, China). *Alternaria mali* Roberts strain was inoculated in potato dextrose agar (PDA) medium and cultured at 28 °C for 5 days. The mycelium was cultured in liquid medium (10 g/L Glucose, 2 g/L Yeast extract fermentation, 0.5 g/L KH_2_PO_4_, 0.5 g/L MgSO_4_·7H_2_O, and 0.5 g/L CaCl_2_) to be able to access the edge. The optimum sugar production conditions were determined to be fermentation at 28 °C with shaking at 120 rpm for 6 days.

### 3.2. AMEP Extraction and Purification

The fermentation liquid leaching filtrate was centrifuged for 15 min at 5000 rpm, the solution was concentrated to an appropriate volume, a 3-times volume of 95% ethanol was added, and the solution was vigorously mixed and left to react overnight at 4 °C. The centrifugation step was repeated to collect the precipitate, which was dissolved in pure water and then centrifuged again to collect the supernatant. The polysaccharide solution was deproteinized by Sevage method [34]. The polysaccharide solution was bleached with D3520 macroporous resin (Nankai University, Tianjin, China) and then lyophilized to obtain AMEP.

The crude polysaccharides were separated on a DEAE-Sepharose Fast Flow ion-exchange chromatography column (1.6 cm × 20 cm, GE Healthcare Life Science, Marlborough, MA, USA), as previously described [35,36]. Elution was performed with Tris-HCl buffer (pH 7.4, Biosharp Technology, Hefei, China) and then with Tris-HCl buffer containing NaCl (0–0.5 M, pH 7.4). The components were collected according to the elution curve, and the two polysaccharides were obtained from two peaks, termed AMEP-1 and AMEP-2 respectively. S-400 HR gel chromatography columns (1.6 cm × 60 cm, GE Healthcare Life Science, Marlborough, MA, USA) were further used for purification of the polysaccharides using distilled water [28,37]. Finally, pure polysaccharides were obtained by lyophilization after 48 h of dialysis using pure water (Biosharp Technology, Hefei, China).

### 3.3. Determination of Purity and Molecular Weight

The purity and average molecular weight of AMEP-1 and AMEP-2 were detected according to a previously described method using a combination of high-performance size exclusion chromatography and multi-angle laser light scattering (HPSEC-MALLS). The chromatographic conditions were as follows: Waters 2695 (Waters, Milford, MA, USA) and OHpak SB-803M chromatograph (Shodex, Tokyo, Japan); Optilab T-rEX refractive index detector (Optilab, Phoenix, Arizona, USA); DAWN HELEOS multi-angle laser light scattering instrument (Wyatt, Santa Barbara, CA, USA); mobile phase: pure water; flow rate: 0.5 mL/min; and column temperature: 25 °C. 

### 3.4. Monosaccharide Composition Analysis

Five milligrams of AMEP-1 and AMEP-2 each were mixed with 2 mol/L trifluoroacetic acid, and hydrolysis was performed at 110 °C for 4 h. Steaming was repeated with methanol until no sourness was achieved. The sample was then washed with 1 mL distilled water and used for pre-column derivatization under the following conditions: mannose (Man) as a monosaccharide control, glucose (Glc), glucuronic acid (GlcA), glucosamine (GlcN), rhamnose (Rha), galactose (Gal), galacturonic acid (GalA), galactosamine (GalN), xylose (Xyl), fucose (Fuc), equimolar mixture of pure water, and standard solution mixture (5 mg/mL). To each 1.5 mL centrifuge tube, 0.3 mol/L NaOH and 0.5 mol/L 1-phenyl-3-methyl-5-pyrazolone (PMP) solution (Sigma-Aldrich, Prefecture, MO, USA) were added to produce the derivative. After neutralization with 0.3 mol/L HCl, chloroform extraction was carried out several times and then the sample was centrifuged to retain the supernatant, which was used for liquid chromatography [31,38,39].

The liquid chromatography conditions were as follows: Eclipse XDB-C18 column (4.6 mm × 150 mm, Agilent, Santa Clara, CA, USA); mobile phase: phosphate buffer/acetonitrile (*v*/*v*) = 82:18; flow rate: 0.8 mL/min; injection volume: 20 μL; column temperature: 30 °C; and UV detector (245 nm, UltiMate 3000, Dionex, Sunnyvale, CA, USA).

The standard curve was drawn by preparing the standard mixture of monosaccharides at different concentrations. The peaks were plotted and analyzed in parallel and compared to the plotted corresponding peak area.

Ultimately, the monosaccharide composition was determined after hydrolyzation of the AMEP-1 and AMEP-2 polysaccharides with the same monosaccharide standard, and the molar ratio of the monosaccharide composition was calculated according to the standard curve.

### 3.5. Infrared Spectrum Analysis

Ten milligrams of AMEP-1 and AMEP-2 each were subjected to compression with a KBr tablet, and scanned with a Fourier-transformation infrared spectrometer at 4000–400 cm^−1^ (Perkin Elmer, Waltham, MA, USA).

### 3.6. Nuclear Magnetic Resonance (NMR) Spectroscopy Analysis

NMR spectroscopy was conducted according to a previously described protocol [36]. In brief, 10 mg each of the AMEP-1 and AMEP-2 samples were dissolved in 0.5 mL D_2_O and then detected on a 500-MHz NMR spectrometer at 25 °C from 4000 scans (Avance-300 Superconducting NMR spectrometer, Bruker, Fällanden, Switzerland).

### 3.7. Cell Culture

The in vitro anti-tumor effects of AMEP-2 were evaluated in the human gastric cancer cell line BGC-823 from the cell bank of the Shanghai Academy of Biological Sciences, Chinese Academy of Sciences (Shanghai, China). The BGC-823 cells were cultured in serum-free RPMI 1640 medium (containing 80 U/mL penicillin and 0.08 mg/mL streptomycin), supplemented with 10 mL fetal bovine serum at 5% CO_2_ and 37 °C to obtain cells in the logarithmic growth phase.

### 3.8. Proliferation Assay

The logarithmic-phase BGC-823 cells were cultured in 96-well culture plates with a 100-μL cell suspension (8 × 10^4^ cells/mL) per well. The plates were incubated for 6–8 h at 37 °C with 100 μL of complete medium per well along with different concentrations of AMEP-2 (0, 200, 400, 600, 800, 1200, 1600, and 2000 μg/mL) for 24, 48, and 72 h. Twenty microliters of 2,5-diphenyltetrazolium bromide (MTT) solution (5 mg/mL; Sigma, St. Louis, MO, USA) were added to each well and the plates were further incubated for 4 h. The supernatant was obtained and mixed with 150 μL dimethyl sulfoxide. The absorbance values of each well were measured with a microplate reader at 490 nm, and the extent of growth inhibition was determined with the following formula:

Growth inhibition rate = (control group A_490nm_ − treatment group A_490nm_/control group A_490nm_) × 100%.

### 3.9. Cell Cycle Detection

The logarithmic phase BGC-823 cells were cultured in 6-well plates and incubated for 12 h at 37 °C. After washing with PBS, 2 mL of medium without serum was added, and the cells were starved for 12 h. A different concentration of AMEP-2 (0, 400, 800, and 1600 μg/mL) was added to each well, and the cells were further incubated for 48 h. The cells were harvested, washed with ice-cold PBS and fixed with 70% ethanol at 4 °C overnight. After centrifugation, the pellets were washed twice with PBS, mixed with propidium iodide (PI) staining solution, and incubated with RNaseA (Keygen Institute of Biotechnology, Nanjing, China) for 30 min at 37 °C in the dark. Eventually, the DNA content and the percentage of cells in each phase of the cell cycle were analyzed in triplicate for each experiment by flow cytometry.

### 3.10. Hoechst 33258 Staining

Hoechst 33258 can penetrate the cell membrane and combines with DNA to produce blue fluorescence after ultraviolet excitation, which can be observed under a fluorescence inverted microscope. Under normal circumstances, the cell nuclear staining is uniform, emitting pale blue fluorescence; however, chromatin condensation and nuclear fragmentation occur after apoptosis along with the formation of apoptotic bodies, which results in bright blue fluorescence. Therefore, this staining method is commonly used for the detection of apoptosis, and was adopted in the present study to detect the effects of AMEP-2 on the apoptosis of gastric cancer cells.

The cell density was adjusted and the cells were cultured as described above in Section 3.9. The cells were fixed with Hoechst 33258 staining reagent (Beyotime Institute of Biotechnology, Shanghai, China) for 15 min in PBS at room temperature (20–25 °C), rinsed three times, lightly stained at room temperature for 15 min, and rinsed three more times in PBS for 5 min each time. Anti-fluorescence quenching liquid was then dropped onto the slides, and fluorescence was observed under an inverted microscope and photographed.

### 3.11. Annexin V-FITC–PI Double Staining

BGC-823 cells were cultured in six-well plates and incubated at 37 °C and treated with AMEP-2. Following treatment, 1 mL of trypsin digestion liquid was added to the original medium to digest the cells, and the cells were collected for apoptosis detection with the Annexin V-FITC cell apoptosis detection kit (Beyotime Institute of Biotechnology, Shanghai, China) according to the manufacturer’s instructions. In brief, Annexin V-FITC and PI staining solutions were combined with liquid from the cell suspension (5–10 million cells), incubated in the dark for 20 min at room temperature, and then placed in an ice water bath. The apoptotic cells were then detected with a FACSVerse^TM^ flow cytometer (BD Biosciences, Franklin Lakes, NJ, USA).

### 3.12. Detection of ROS

The generation of ROS was detected with the fluorescent probe DCFH-DA according to the instructions of the ROS detection kit (Beyotime Institute of Biotechnology, Shanghai, China). Logarithmic BGC-823 cells were treated with different concentrations of AMEP-2 (0, 400, 800, and 1600 μg/mL) for 48 h. After removal of the cell culture medium, 1 mL of diluted DCFH-DA solution was added and the cells were incubated at 37 °C for 20 min. Following incubation, the dishes were washed with 1 mL PBS to remove the loading probe, placed under a laser-scanning confocal microscope, and photographed.

BGC-823 cells treated with EPS were collected for the detection of ROS by flow cytometry. In brief, BGC-823 cells were collected by trypsin digestion solution (without EDTA). The cells were placed in 1 mL of diluted DCFH-DA solution, mixed, and incubated for 20 min. After the incubation, the cells were washed with PBS three times, and as much of the unloaded DCFH-DA probe was removed as possible. The cell suspension was formed by adding 500 μL PBS, and flow cytometry was used to detect the ROS generated at an excitation wavelength of 488 nm and an emission wavelength of 525 nm.

### 3.13. Detection of Mitochondrial Membrane Potential

The logarithmic-phase BGC-823 cells were adjusted and cultured as described in Section 3.9, and treated with the respective concentrations of AMEP-2. The cell culture medium was removed, and then the cells were washed with PBS. One milliliter of trypsin digestion solution (containing EDTA) was added to digest the cells and then removed. The culture medium was refreshed, and the cells were collected by centrifugation, and counted after adding PBS to the cell suspension. Mitochondrial membrane potential was detected with the fluorescent probe JC-1. At a high level of mitochondrial membrane potential, JC-1 polymerizes in the matrix resulting in red fluorescence, whereas with low membrane potential, JC-1 remains in the monomer form and shows green fluorescence. Therefore, changes from red to green fluorescence of JC-1 could reflect a decrease in the mitochondrial membrane potential. 

JC-1 staining working fluid and buffer (1×) were prepared from the kit (Beyotime Institute of Biotechnology, Shanghai, China). In brief, 10,000–600,000 cells were added to 0.5 mL culture medium to make the cell suspension, and 0.5 mL JC-1 staining fluid was added and incubated at 37 °C for 20 min. The samples were then washed with JC-1 buffer (1×) twice and analyzed by flow cytometry.

### 3.14. Statistical Analysis

All data are expressed as the mean value ± standard deviation, and were statistically analyzed with SPSS 13.0 statistical software (IBM, New York, NY, USA). The *t*-test was used when the variance was homogeneous, and the *t*’-test was used when the variance was not uniform between groups. A *p* value of less than 0.05 was considered to indicate a statistically significant difference.

## 4. Conclusions

In this study, an extracellular polysaccharide from *Alternaria mali* Roberts (AMEP) was extracted, and then two fractions, named AMEP-1 and AMEP-2, were obtained for further research. MALLS analysis of AMEP-1 and AMEP-2 revealed an average molecular weight of 3.861 × 10^4^ Da and 4.689 × 10^4^ Da, respectively. The monosaccharide composition analysis showed two kinds of polysaccharides containing mannose, glucose, and galactose in different proportions. The results of IR analysis showed that both AMEP-1 and AMEP-2 were polysaccharides. NMR analysis showed that the linking mode of AMEP-1 and AMEP-2 is Man*p*-(1→4) and Glc*p*-(1→6), the branched chains are connected to the main chain through O-6. AMEP-2 showed a notable inhibitory effect on the proliferation of BGC-823 cells in vitro, in a time- and concentration-dependent manner. AMEP-2 induced cell cycle arrest and ROS generation, and reduced mitochondrial membrane potential, thus exerting an antitumor effect by inducing apoptosis in BGC-823 cells. An in-depth study of AMEP-2 might help in its development as a novel antitumor agent.

## Figures and Tables

**Table 1:** Experimental Results of MALLS for AMEP-1 and AMEP-2.

Group	Polydispersity		Molar Mass Moments (g/mol)			Root Mean Square Radius Moments (nm)		
	Mw/Mn	Mz/Mn	Mn	Mw	Mz	Rn	Rw	Rz
AMEP-1	1.492	2.858	2.588 × 10^4^	3.861 × 10^4^	7.395 × 10^4^	15.2	12.5	11.6
AMEP-2	1.849	3.47	2.536 × 10^4^	4.689 × 10^4^	8.798 × 10^4^	35.5	30.2	22.7

**Table 2:** Cytotoxic effects of AMEP-2 on BGC-823 cells ($\bar{x}$ ± s, *n* = 6, %, * *p* < 0.05 vs. control, ** *p* < 0.01 vs. control).

Group	Concentration μg·mL^−1^	Inhibitory Rate of Cell Growth		
		24 h	48 h	72 h
Control	0	0 ± 6.32	0 ± 5.55	0 ± 6.68
AMEP-2	200	5.12 ± 2.82	6.69 ± 4.12	8.5 ± 2.89
	400	8.65 ± 2.15 *	7.82 ± 5.54	10.86 ± 3.92 *
	600	9.63 ± 1.53 **	11.84 ± 6.61	24.25 ± 1.3 *
	800	13.37 ± 3.52 **	20.48 ± 4.1 **	27.37 ± 5.13 *
	1200	14.48 ± 5.98 **	26.84 ± 6.33 **	38.42 ± 3.67 **
	1600	19.6 ± 1.65 **	34.34 ± 4.59 **	43.18 ± 2.62 **
	2000	25.05 ± 2.74 **	35.86 ± 2.14 **	46.84 ± 2.5 **
